# Green Synthesis of Metal-Organic Framework Bacterial Cellulose Nanocomposites for Separation Applications

**DOI:** 10.3390/polym12051104

**Published:** 2020-05-13

**Authors:** Radwa M. Ashour, Ahmed F. Abdel-Magied, Qiong Wu, Richard T. Olsson, Kerstin Forsberg

**Affiliations:** 1 Department of Chemical Engineering, KTH Royal Institute of Technology, 100 44 Stockholm, Sweden; 2 Nuclear Materials Authority, P.O. Box 530, ElMaadi, Cairo 11381, Egypt; 3 Department of Fibre and Polymer Technology, KTH Royal Institute of Technology, 100 44 Stockholm, Sweden; qiongwu@kth.se

**Keywords:** bacterial cellulose, metal organic framework, nanocomposite, adsorption

## Abstract

Metal organic frameworks (MOFs) are porous crystalline materials that can be designed to act as selective adsorbents. Due to their high porosity they can possess very high adsorption capacities. However, overcoming the brittleness of these crystalline materials is a challenge for many industrial applications. In order to make use of MOFs for large-scale liquid phase separation processes they can be immobilized on solid supports. For this purpose, nanocellulose can be considered as a promising supporting material due to its high flexibility and biocompatibility. In this study a novel flexible nanocellulose MOF composite material was synthesised in aqueous media by a novel and straightforward in situ one-pot green method. The material consisted of MOF particles of the type MIL-100(Fe) (from Material Institute de Lavoisier, containing Fe(III) 1,3,5-benzenetricarboxylate) immobilized onto bacterial cellulose (BC) nanofibers. The novel nanocomposite material was applied to efficiently separate arsenic and Rhodamine B from aqueous solution, achieving adsorption capacities of 4.81, and 2.77 mg g^−1^, respectively. The adsorption process could be well modelled by the nonlinear pseudo-second-order fitting.

## 1. Introduction

Since the advent of the industrial revolution, dumping of large amounts of industrial waste including dyes and toxic metal ions has contributed to the serious issue of water pollution [1]. Consequently, various techniques for the removal of organic dyes and toxic metal ions from aqueous solutions, including adsorption [2,3,4], chemical precipitation [5], ion exchange [6], and membrane separation [7] have been evaluated. Amongst these, adsorption is proven to be an effective and convenient method for water purification due to the ease of operation and the low cost. However, for nanoadsorbents, complicated and tedious high-speed centrifugation or separation of the adsorbent using filtration is required, hindering the extensive application of such adsorbents. Therefore, development of novel materials for water treatment is of great interest. 

Metal‒organic frameworks (MOFs) are porous hybrid materials composed of metal ions bridged by polydentate organic ligands. Since the synthesis of MOF-5 reported by Yaghi and co-workers (1999) [8], MOFs have received significant attention due to their high crystallinity, large surface areas, thermal stability and unique porosity. MOF applications are numerous, including catalysis [9,10,11], drug delivery [12], gas storage [13,14,15], chemical sensing [16], and separation [17,18,19]. The properties of the MOF structures can be easily tuned by selecting different metal ions and bridging organic polydentate ligands, and the design and preparation of new MOFs into various structures is still of great interest [20]. However, the handling and processing of MOFs are also challenging due to the crystalline nature of the MOFs, making them brittle and fragile as inorganic materials [21,22]. To tackle this problem, efforts have been made to entrap MOFs onto various substrates, thus realizing a combination of their advantages, while circumventing, and to different extents addressing the MOF’s shortcomings. Various MOF composites have been successfully prepared via direct deposition of MOF particles on solid substrate surfaces [23]. However, although such synthetic protocols produce a new types of functional materials, their applications are limited by their constrained morphologies and synthetic protocols. Usually, substrate surface modifications are needed to increase the MOF loading and conditions must be applied that in many cases involve environmentally hazardous reagents [24]. 

Membrane adsorption can be applied to separate metal ions in larger scale. For this purpose, a column is used that is packed with a short stack of porous membranes with a large diameter, to avoid high pressure drops. In membrane adsorption fibres can act as an adsorbing medium. One advantage of this technology is that process flow rates can be orders of magnitudes higher than for packed beds, without elevated pressure. The challenge is to achieve a comparable adsorption capacity to that of packed beds. However, by using functionalised nanofibers the adsorption capacities can even exceed packed bed resins due to the very high surface area of the fibres. Refined low-cost biopolymers such as nanocellulose can here be considered as a promising supporting material due to its very interesting properties, such as high chemical purity and crystallinity, flexibility and biocompatibility [25]. Furthermore, nanocellulose is the most abundant and renewable green biopolymer and a sustainable raw material. Bacterial cellulose (BC) fibrils are one of the most frequently reported nanocellulose types. It is produced by cultivating *Acetobacter xylinum* in the presence of sugar [26]. BC nanofibrillar cellulose shows great promise to be used as a substrate since it can be generated in high yield from its natural source containing >70 wt% crystalline cellulose. It also shows high aspect ratio due to its fibrillar length reaching up to several micrometers while at the same time providing average fiber diameters of 20–100 nm [27]. To date, only a few studies have reported integration of MOFs onto nanocellulose. Matsumoto and co-workers reported the successful growth of MOFs (up to 44% loading) at carboxyl groups on crystalline TEMPO-oxidized cellulose nanofibers and prepared densely packed films coated on a filter paper, which demonstrated high gas separation selectivity [28]. Zhu and co-workers have developed a strategy for combining MOFs and cellulose nanocrystals into a highly functional aerogel for separation applications, where the MOF loading can be easily tailored by changing the initial ratio of the components [29]. Recently, Au-Duong and co-workers successfully demonstrated that a flexible nanocomposite pellicle combining imidazolate framework-8 (ZIF-8) and wet BC could be simply synthesized when polydopamine surface coating on cellulose nanofibers is applied in advance [30].

Herein, we report a new, straightforward, environmentally friendly in situ green strategy to integrate MOF Material Institute de Lavoisier-100(Fe), Fe-BTC (alternatively known as MIL-100(Fe); BTC = 1,3,5-benzenetricarboxylate) [31] into BC in aqueous media. This method makes it possible to synthesize cellulose-based metal organic framework nanocomposite material without using any chemical modifier. The synthetic approach provides a general strategy to prepare such nanocomposites for various applications. The synthesized MIL-100(Fe)@BC nanocomposite not only maintained high micro/ mesoporosities of the MIL-100(Fe) particles, but also combined flexibility and shapeability of the BC crystal support. This combination produces a shapeable, low-cost, chemically inert and scalable product that can be used in various applications, for example, water purification. The synthesized hybrid MIL-100(Fe)@BC nanocomposite was evaluated in water purification as an efficient adsorbent for the removal of arsenic As(III), and Rhodamine B. 

## 2. Materials and Methods

*Materials*: Iron(III) chloride hexahydrate (≥98%), 1,3,5-benzenetricarboxylate (98%) were purchased from VWR (Stockholm, Sweden). Rhodamine B (≥95%) was purchased from *Sigma-Aldrich* (Stockholm, Sweden). Arsenic pure single-element standard (1000 mg L^−1^) was purchased from PerkinElmer (Stockholm, Sweden). All chemicals were used as received without further purification. Deionized water (resistivity: 18.2 MΩ/cm) was used to prepare aqueous solutions. The bacterial cellulose nanofibrils were extracted from a uniformly grown bacterial cellulose pellicle, using 1 L aqueous sulphuric acid solution containing 30 vol% of reagent grade acid (Merck KGaA, Stockholm, Sweden) under stirring at 60 ± 0.5 °C (300 rpm). The total time for the extraction was 7 h, i.e., until no visible cellulose pieces were apparent and the solution had acquired an even beige color. The supernatant was decanted after a first isothermal centrifugation at 10,000 rpm for 10 min and replaced with fresh MilliQ water. The procedure was repeated twice until a neutral pH was obtained. The yield of the extraction was ca. 60 wt% based on the dry weight of the bacterial cellulose. The surface area of the bacterial cellulose nanofibrils was approximated to ca. 159 m^2^ g^−1^ from size distributions and counting of a minimum of 500 fibrils deposited on TEM grids. The approximated surface area was in relatively good agreement with the surface area of 189 m^2^ g^−1^ reported by Roman et al. [32] while larger than the experimental value of 103 m^2^ g^−1^ for dry bacterial cellulose fibrils by Olsson et al. [26]. 

*In situ one-pot synthesis of MIL-100(Fe)@BC*: Iron(III) chloride hexahydrate (164 mg) was added to 50 mL of well-sonicated BC (2.0 wt% dry contents) and the reaction mixture was refluxed at 100 °C under mechanical stirring (350 rpm). After 30 min, different concentrations of 1,3,5-benzenetricarboxylate were dissolved in 4 mL of deionized water and added to the reaction mixture, which was kept for an additional 30 min at 100 °C. The final suspension was centrifuged and washed 3 times with acetone followed by deionized water. The product was placed in a plastic tube that was directly frozen by immersing the tube in liquid N_2_ followed by freeze-drying to obtain the final hybrid nanocomposite MIL-100(Fe)@BC, that was activated at 100 °C for 12 h before being used as an adsorbent. 

*Adsorption*: The adsorption experiments were carried out at room temperature. Adsorption studies were carried out by soaking MIL-100(Fe)@BC (0.18 g) in aqueous solution containing different contaminants (20 mg L^−1^ of As(III) (50 mL) and 10 mg L^−1^ of Rhodamine B (40 mL)) for a certain amount of time. After adsorption, the MIL-100(Fe)@BC nanocomposite was separated from the aqueous solution, and the dye concentration was analyzed by UV-vis based on a calibration curve prepared from solutions with known contaminant concentration at the maximum wavelengths (554 nm). The concentration of As(III) in the supernatant was determined by inductively coupled plasma atomic emission spectroscopy (ICP-OES, Waltham, MA, USA). The adsorption experiments were performed in duplicate/triplicate and the average values of total adsorption are reported. The adsorption capacity (*q_t_*, mg g^−1^) and the removal % at time *t* were determined using Equations (1) and (2).

(1) $$q_{t}=\frac{\left(C_{0}-C_{t}\right)V}{m}$$

(2) $$\mathrm{Removal}\;\left(\%\right)=\frac{C_{0}-C_{t}}{C_{0}}\times 100$$

where *C_0_* and *C_t_* (mg L^−1^) are the initial concentration and concentration at time t in aqueous solution, respectively, *V* (L) is the volume of the aqueous phase, and *m* (g) is the mass of the MIL-100(Fe)@BC nanocomposite. The kinetics of the adsorption process was investigated by fitting the nonlinear forms of pseudo-first order and pseudo-second order models to the data using Equations (3) and (4).

(3) $$q_{t}=q_{e}\left(1-e^{-k_{1}t}\right)$$

(4) $$q_{t}=\frac{k_{2}q_{e}^{2}t}{1+k_{2}q_{e}t}$$

where *k_1_* (min^−1^) and *k_2_* (g mg^−1^ min) are the pseudo-first-order and pseudo-second-order rate constants of adsorption, *q_t_* and *q_e_* (mg g^−1^) are the adsorption capacity at a given time *t* and at equilibrium, respectively.

*Characterization*: Scanning electron microscopy (SEM) measurements were conducted on a Hitachi S-4800 microscope (Tokyo, Japan) using accelerating voltages of 2 kV to 10 kV. High resolution transmission electron microscopy (HRTEM) was performed at 90 K using a JEOL-2011 (Tokyo, Japan) at 200 kV. Powder X-ray diffraction was conducted on a Bruker D8 advance X-ray diffractometer (Madison, WI, USA). The concentration of the contaminated water was determined using an UV-vis (ultraviolet-visible) spectrophotometer (DR 3900, Hach, Stockholm, Sweden). The total concentrations of arsenic ions were determined by ICP-OES (Thermo Fisher iCAP 7400, Waltham, MA, USA). The pH value of the solution was measured by a pH-meter (ORION Star A211, Thermo ScientificTM, Waltham, MA, USA). Thermogravimetric analysis (TGA) was performed under a nitrogen atmosphere from 25 °C to 700 °C with a heating rate of 5 °C min^−1^ using a thermogravimetric analyzer (TGA/SDTA 851e, Mettler Toledo, Mississauga, ON, Canada).

## 3. Results and Discussion

**Scheme 1:**
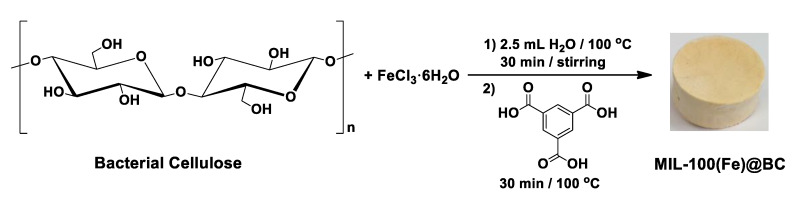
General procedure for preparation of the MIL-100(Fe)@BC nanocomposite.

**Figure 1:**
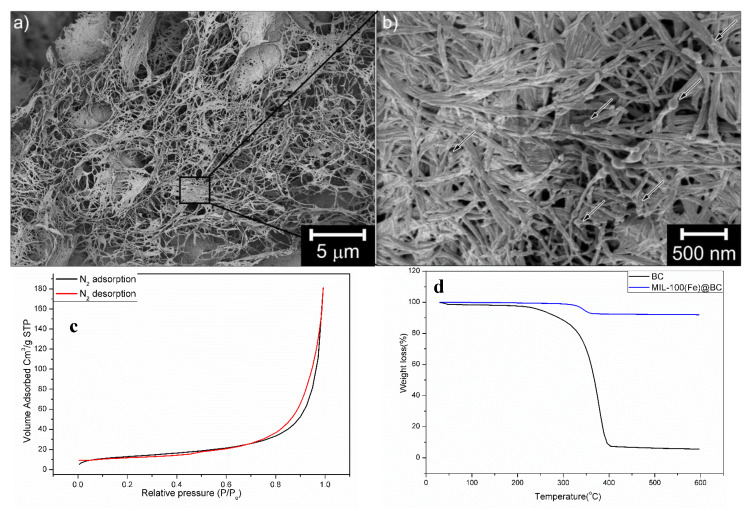
(**a**) and (**b**) SEM image of hybrid MIL-100(Fe)@BC nanocomposite (BTC/Fe(III) = 120), (**c**) Brunauer‒Emmett‒Teller (BET) analysis of MIL-100(Fe)@BC (BTC/Fe(III) = 120), and (**d**) thermogravimetric analysis (TGA) of bacterial cellulose (BC) and MIL-100(Fe)@BC.

The hybrid MIL-100(Fe)@BC nanocomposites were synthesized by mixing BC and Fe(III) under refluxing conditions for 30 min followed by the addition of BTC at different molar ratios of BTC/Fe(III), as shown in Scheme 1. When Fe(III) ions are added to the BC solution in the preparation of the MIL-100(Fe)@BC nanocomposite, complexation interaction will take place between the Fe(III) ions and the BC crystals hydroxyl groups, and the Fe(III) ions concentration will increase in the vicinity of the fibrillar BC crystals surface. Once the ligand precursor BTC was added to the reaction mixture, the binding Fe(III) ions would participate to form MIL-100(Fe) crystals, that grew gradually on the BC network that became partially immobilized as the inorganic condensations occurred. At a BTC/Fe(III) molar ratio of 25, aggregation of MIL-100(Fe) crystals with particles of average size > 400 nm appeared on the surface of the BC and the nanofibers could barely be observed after freeze-drying (Figure S1). However, as the BTC/Fe(III) molar ratio increased to 120, MIL-100(Fe) particles with smaller sizes were uniformly observed on the surface of the BC. These were stable enough to persist during the freeze-drying and form the hybrid nanocomposite MIL-100(Fe)@BC (Figure 1a,b). 

An increased ligand/metal ion molar ratio resulted in the formation of smaller MOF particle sizes on the surface of BC. The MOF size reduction upon increasing the ligand/metal ion molar ratio has also been observed by other researchers [29]. Such behaviour can be explained by the high supersaturation driving force at higher ligand/metal ion ratios, leading to high nucleation in the early stage of MOF synthesis and the formation of small MOF crystals [33]. The resultant hybrid MIL-100(Fe)@BC nanocomposite was flexible, ultralight and mechanically robust enough to be easily processed and further evaluated without any loss or damage of the MOFs structural integrity. 

The powder X-ray diffraction patterns for BC and 100(Fe)@BC nanocomposite confirmed the formation of MIL-100(Fe) within the BC crystal network (Figure S2). Brunauer‒Emmett‒Teller (BET) analysis using N_2_ sorption was also employed to determine the surface area of the prepared MIL-100(Fe)@BC nanocomposite (Figure 1c). The BET surface area of the MIL-100(Fe)@BC nanocomposite is 47.13 ± 0.15 m^2^/g.

Figure 1d shows the thermogravimetric analysis (TGA) of the MOF-cellulose hybrid material and the BC. A gradual decrease in weight of ca. 0.3 wt% occurred up to ca. 180 °C, which was synonymous with structurally bonded water entrapped in the porous material. Further condensation and densification (ca. 1 wt%) of the material occurred from ca. 180 °C until the degradation temperature of the cellulose was reached at ca. 350 °C. The sharp weight loss to 91.8 wt% (of the materials original weight) at 350 °C could thus be correlated to a total loading of bacterial cellulose equivalent with ca. 8 wt%. At temperatures above 400 °C, degradation and evaporation of carbon residuals occurred. The density of the entire cylindrical sample (Scheme 1) could further be derived from the volume of the sample and its weight. The low density of MIL-100(Fe)@BC was 47.8 mg cm^−3^, implying that the nanocomposite was very porous with a large accessible surface area. 

**Figure 2:**
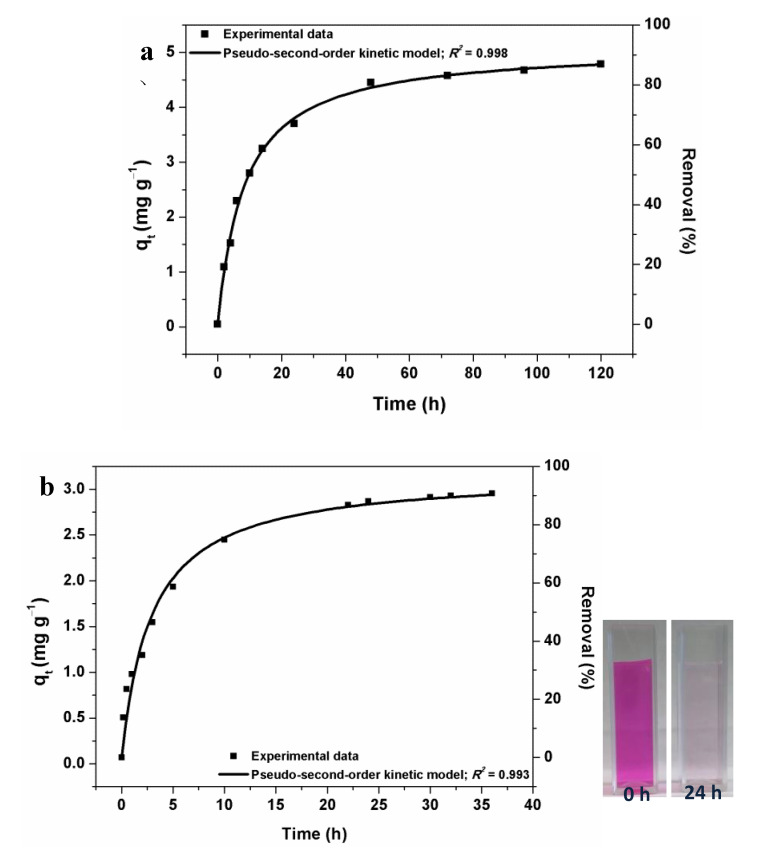
Adsorption capacity versus time, and the nonlinear pseudo-second-order fitting for the adsorption of As(III) (**a**); and Rhodamine B (**b**) on MIL-100(Fe)@BC nanocomposite, respectively. The inset shows a photograph of the Rhodamine B aqueous solutions before and after the adsorption process.

Owing to their high specific surface area, MOFs are intended for various applications [34]. Herein, we evaluate the synthesized MIL-100(Fe)@BC nanocomposite in water purification to demonstrate the adsorption ability of MOF-cellulose materials as interpenetrating networks with an integrated cellulose phase. Arsenic is recognized as one of the most hazardous metal ions in drinking water and is listed by the World Health Organization among the top 10 major public health concerns. In this context, the adsorption capacity of the MIL-100(Fe)@BC nanocomposite was tested for the removal of As(III) from aqueous solutions. In the tests, a small MIL-100(Fe)@BC nanocomposite (0.18 g) was soaked into 50 mL of an aqueous solution containing As(III) (20 ppm) (See Experimental Section for more details). The adsorption capacities (q_t_ in mg g^−1^) and removal (%) of As(III) at different times (t) are shown in Figure 2a. High adsorption capacity for As(III) adsorption onto MIL-100(Fe)@BC nanocomposite was observed (removal efficiency ≈ 85% within 72 h).

Two kinetic models, the pseudo-first-order and pseudo-second-order models, were used to investigate the mechanism of As(III) adsorption on the MIL-100(Fe)@BC nanocomposite (Figure S3a and Figure 2a, respectively). The nonlinear pseudo-second-order kinetic model fitted well to the experimental data (Figure 2a), and the experimental *q_t,exp_* values and the calculated *q_t,cal_* values obtained from the pseudo-second-order kinetic model are in good agreement (Table S1). The calculated values of the pseudo-second-order parameters are listed in Table S1. The fitting results indicate that the BC network is not hindering the accessibility of the MIL-100(Fe) pores and most of the MIL-100(Fe) particles are functional in the adsorption of As(III) onto the MIL-100(Fe)@BC nanocomposite as a chemical process via surface complexation [35]. The total concentration of Fe in the solution was measured by ICP-OES after the adsorption experiments in order to investigate the stability of the material. No Fe was detected, indicating a high stability of the synthesized MIL-100(Fe)@BC nanocomposite under the employed conditions. 

Removal of organic dyes from aqueous solution is another significant challenge in the field of water purification. Among several organic dyes, Rhodamine B was chosen for the present study due to its extensive use as a colorant in the textile and food industries [36]. Rhodamine B was used as a model tracer to monitor the adsorption capacity and kinetics for adsorption onto the MIL-100(Fe)@BC nanocomposite. Herein, a small amount of the nanocomposite MIL-100(Fe)@BC (0.18 g) was added to 40 mL of an aqueous solution containing Rhodamine B (10 ppm), and the dye concentration in the solution at certain time was determined by UV-Vis (see Experimental Section for more details). The adsorption experiments were carried out at room temperature. The adsorption capacity (*q_t_*) and removal percentage of Rhodamine B as a function of time (*t*) are shown in Figure 2b. The adsorption capacities for Rhodamine B increased with increasing time, and reached equilibrium within 24 h, achieving a removal percentage of 85%. The high adsorption capacity of the MIL-100(Fe)@BC nanocomposite results from the hierarchical porosity and small MIL-100(Fe) crystals integrated within the BC network, which offer a high number of external surface sites for the adsorption.

The kinetics for Rhodamine B adsorption onto the MIL-100(Fe)@BC nanocomposite was studied using pseudo-first-order and pseudo-second-order models (Figure S3b and Figure 2b, respectively). The obtained experimental data for the adsorption process, determined by UV-Vis at 554, fitted very well with the pseudo-second-order kinetic model (Figure 2b), which indicate that the adsorption was controlled by intraparticle diffusion [37]. The calculated values of the pseudo-second-order parameters are listed in Table S1. After soaking the MIL-100(Fe)@BC nanocomposite with the Rhodamine B aqueous solution, the pink dye solution gradually faded into colorless over time, and its UV-Vis maximum absorption beak at 554 nm reduced significantly (Figure S4). The inset in Figure 2b visually confirms the color change of Rhodamine B aqueous solution before and after the adsorption process by MIL-100(Fe)@BC nanocomposite. The adsorption mechanism can be attributed to the electrostatic interactions between MIL-100(Fe)@BC nanocomposite and Rhodamine B [38]. 

A comparison of the obtained results for As(III) and Rhodamine B removal using MIL-100(Fe)@BC nanocomposite with other reported systems is summarized in Table 1.

## 4. Conclusions

A MIL-100(Fe)@BC nanocomposite was synthesized by an environmentally friendly method using water as solvent. The bacterial cellulose (BC) acted as structural support during the lyophilisation of the MIL-100(Fe), which resulted in a flexible and light weight material suitable for membrane adsorption processes. It was demonstrated that the size of the loaded MIL-100(Fe) particles on the BC can be tailored by changing the initial ratio of MIL-100(Fe) precursors. The synthesized nanocomposite is efficient in removal of As(III) and Rhodamine B from aqueous solutions. The kinetic studies revealed that the adsorption of As(III) and Rhodamine B was best fitted to the pseudo-second order model. The synthetic approach provides a simple strategy to prepare the MOFs@BC nanocomposites for various applications, in particular for water purification. Further studies on how to immobilize other types of MOFs using BC could be explored.

## Figures and Tables

**Table 1:** Comparison of As(III) and Rhodamine B removal with other reported adsorbents.

Adsorbents	q_e_ (mg g^−1^)	Ref.
**As(III) Removal**		
MIL-53(Al)-graphene oxide	65.0	[39]
Surfactant-modified montmorillonite	1.48	[40]
Magnetic pinecone biomass	18.02	[41]
Zn-MOF	49.50	[42]
MIL-100(Fe)	120	[35]
MIL-100(Fe)@BC	4.81	This work
**Rhodamine B Removal**		
Hypercross-linked polymeric adsorbent	2.1	[43]
Mango leaf power	3.31	[44]
Zn-MOF	3.750	[45]
Fe_3_O_4_/MIL-100(Fe)	28.36	[38]
MIL-100(Fe)@BC	2.77	This work

## Supplementary Materials

The following are available online at https://www.mdpi.com/2073-4360/12/5/1104/s1, Figure S1: Photograph (inset) and TEM image of MIL-100(Fe)@BC nanocomposite (BTC/Fe(III) = 25), Figure S2: XRD patterns of a) BC and b) MIL-100(Fe)@BC nanocomposite (BTC/Fe(III) = 120), Figure S3: Plots of pseudo-first-order kinetics models for the adsorption of As(III) (a) and Rhodamine B (b) using MIL-100(Fe)@BC nanocomposite (BTC/Fe(III) = 120), Figure S4: UV-Vis spectra for the adsorption of Rhodamine B using MIL-100(Fe)@BC nanocomposite (BTC/Fe(III) = 120) at different time, and Table S1: Kinetic parameters for the adsorption of As(III) and Rhodamine B using MIL-100(Fe)@BC nanocomposite.
